# Extended Graphite Supported Flower-like MnO_2_ as Bifunctional Materials for Supercapacitors and Glucose Sensing

**DOI:** 10.3390/nano11112881

**Published:** 2021-10-28

**Authors:** Han-Wei Chang, Chung-Li Dong, Yan-Hua Chen, Yuan-Zhang Xu, Tzu-Chi Huang, Song-Chi Chen, Feng-Jiin Liu, Yin-Hung Lai, Yu-Chen Tsai

**Affiliations:** 1 Department of Chemical Engineering, National United University, Miaoli 360302, Taiwan; U0714022@gm.nuu.edu.tw (Y.-H.C.); U0714139@gm.nuu.edu.tw (Y.-Z.X.); j927528@gmail.com (T.-C.H.); chen31423@yahoo.com.tw (S.-C.C.); liu@nuu.edu.tw (F.-J.L.); 2 Pesticide Analysis Center, National United University, Miaoli 360302, Taiwan; 3 Department of Physics, Tamkang University, Tamsui, New Taipei City 25137, Taiwan; cldong@mail.tku.edu.tw; 4 Institute of Food Safety and Health Risk Assessment, National Yang Ming Chiao Tung University, Taipei 11221, Taiwan; 5 Department of Chemical Engineering, National Chung Hsing University, Taichung 40227, Taiwan

**Keywords:** MnO_2_/extended graphite, supercapacitors, glucose sensing

## Abstract

A simple, efficient, and cost-effective extended graphite as a supporting platform further supported the MnO_2_ growth for the construction of hierarchical flower-like MnO_2_/extended graphite. MnO_2_/extended graphite exhibited an increase in sp^2^ carbon bonds in comparison with that of extended graphite. It can be expected to display better electrical conductivity and further promote electron/ion transport kinetics for boosting the electrochemical performance in supercapacitors and glucose sensing. In supercapacitors, MnO_2_/extended graphite delivered an areal capacitance value of 20.4 mF cm^−2^ at 0.25 mA cm^−2^ current densities and great cycling stability (capacitance retention of 83% after 1000 cycles). In glucose sensing, MnO_2_/extended graphite exhibited a good linear relationship in glucose concentration up to about 5 mM, sensitivity of 43 μA mM^−1^cm^−2^, and the limit of detection of 0.081 mM. It is further concluded that MnO_2_/extended graphite could be a good candidate for the future design of synergistic multifunctional materials in electrochemical techniques.

## 1. Introduction

Over the years, fossil fuel consumption and public health problems have posed serious issues, including global warming and substantial human diseases, that have caused significant negative economic and human health impacts. The relationship expressed above indicated that key technological changes and suitable implementation strategies might be necessary for promoting opportunities to reduce fossil fuel dependency, and, simultaneously, implement health promotion/disease prevention. Supercapacitors serve as intermediate energy storage systems between traditional capacitors and batteries, due to their simple construction, short charging time, safety, high power density, and excellent lifetime, which could replace fossil fuel use to meet energy requirements for reducing the fossil fuel energy consumption and further relaxing environmental pollution.

World-class public health is another globally important issue and challenge. The International Diabetes Federation (IDF) estimated that the number of people worldwide with diabetes (aged 20–79 years) would increase to 700 million in 2045 [1]. In Taiwan, diabetes is the fifth leading cause of death (data obtained from Ministry of Health and Welfare in Taiwan) [2]. Clearly, a fast, simple, efficient, and cost-effective approach needs to be developed in order to monitor blood glucose, allowing for improving glycemic control. Previous studies demonstrated that electrochemical techniques with moderate cost, instrumental simplicity, high portability, and suitable performance showed promise in a diverse range of applications such as solar cells, batteries, sensors, and supercapacitors, and hence provided more realistic solutions to address both the energy and public health challenges.

The choice of suitable electrode materials, which will be used to build novel high-performance electrodes in future electrochemical applications, is critical in the modeling and optimization of electrochemical devices. MnO_2_ nanostructures with well-controlled morphology and crystal phase are expected to exhibit improved electrochemical performance when being used in supercapacitors and electrochemical sensing. More advantages for the use of MnO_2_ as the electrode material were explained in previous reports. Yang et al. reported that MnO_2_ had great advantages in terms of high theoretical specific capacity (up to 1370 F g^−1^), low cost, natural abundance, and multiple crystallographic phases to be one of the most promising candidates for supercapacitors and glucose sensing. The structural transformation of multiple MnO_2_ polymorphic forms composed of MnO_6_ octahedrons by edge and/or corner sharing made differences in the intrinsic channel dimensions of MnO_2_ to possess different electrochemical activities. MnO_2_ based composites for supercapacitor applications with enhanced capacitive behaviors were attributed to electrochemically induced polymorphic phase and structural transformations of MnO_2_, thus affecting the surface area and interfacial resistance [3]. Ponnusamy et al. also reported that MnO_2_ based materials as a result of electrocatalytic effect of Mn^3+^/Mn^4+^ redox couples had excellent electrocatalytic oxidation ability for glucose in the alkaline environment. The result implied that the MnO_2_ based materials could be used as electrochemical electrodes, and they exhibited great excellent performance in electrochemical glucose sensing [4].

However, MnO_2_ based materials possessed inherently low conductivity, which might restrict their practical use in electrochemical techniques [5]. The conductive carbon materials (such as graphite, carbon nanotubes, and graphene, etc.) serving as a conductive component could possibly form the conducting interconnection of carbon materials/MnO_2_ nanocomposites, allowing fast ions and electrons transporting inside interior space of MnO_2_ pore/channel structure, thus dramatically enhancing the electrocatalytic activity [6,7]. Many efforts described that carbon materials served mainly as a conductive component that provided a platform to integrate MnO_2_ pore/channel structure in constructing hierarchical carbon materials/MnO_2_ nanocomposites with three-dimensional (3D) interconnections. It could be expected that MnO_2_ integrated within carbon materials generated a synergistic effect, which would improve electrocatalytic activity for electrochemical multifunctional applications. Several groups of researchers reported that the synthesis of carbon materials/MnO_2_ nanocomposite with enhanced electrochemical activity showed effective characterization in electrochemical applications (supercapacitors and sensors). The improvement in electrochemical performance could be mainly attributed to the synergistic effect between carbon materials and MnO_2_ that tightly connected to obtain dense hierarchical interconnected architectures in shortening the ions diffusion path and facilitating the charge transport [8,9,10].

Among carbon materials, simple, efficient, cost effective, and ecofriendly graphite made it an ideal substrate to grow MnO_2_ for generating carbon materials/MnO_2_ interconnected architectures, and had its own advantages to increase a wide range of industrial-scale production in electrochemical applications. Despite these advantages, pristine graphite had a lower specific surface area, thus causing a lower electrochemical performance. The surface modifications of activated graphite could be obtained by various activation techniques to provide larger specific surface areas at the optimum activation parameters for future enhancement of electrochemical performance. Kabir et al. also reported an electrochemically induced activation in aqueous electrolyte to exfoliate graphite to make surface functionalization. Activation could facilitate the formation of activated regions to improve the specific surface area that ensured excellent surface functional group coverage on graphite surfaces [11]. Chang et al. reported that graphite was efficiently activated by CO_2_ treatment to change the surface area and surface functional groups, leading to low internal resistance and improved charge collection ability, suggesting the enhanced electrochemical behavior of graphite electrodes [12]. Some previous studies also demonstrated that surface modifications of carbon materials led to an increase in polarity of the surface layer, which facilitated the strong interactions to form the strong covalent interfacial bonding with selected substances for the formation of carbon materials/metal nanocomposites, and further highlight its capability to induce the application of a novel electrochemical-based techniques [13,14,15,16].

In this work, the formation of extended graphite through electrochemically activated treatment of graphite as a supporting platform further supports the formation of MnO_2_ for the construction of hierarchical flower-like MnO_2_/extended graphite through spontaneous redox reaction in the KMnO_4_ solution. MnO_2_/extended graphite are anticipated to serve as bifunctional materials for supercapacitors and glucose sensing. This work extends the understanding of electrocatalytic reaction mechanisms between the synergistic effect of MnO_2_ and extended graphite for further electrochemical multifunctional applications in supercapacitors and glucose sensing.

## 2. Materials and Methods

### 2.1. Reagents

Potassium permanganate (KMnO_4_), sodium hydroxide (NaOH), sodium sulfate (Na_2_SO_4_), glucose, carbon black graphite powder, and terpineol were purchased from Sigma-Aldrich (St. Louis, MO, USA). All aqueous solutions were prepared using deionized water (DI water) through a Milli-Q water purification system (Millipore, Burlington, MA, USA). All chemicals were used without further purification.

### 2.2. Preparation of MnO_2_/Extended Graphite Nanocomposite

To obtain a series of extended graphite with different activation degrees, the graphite paste electrodes (3 mm diameter) were immersed in 0.2 M HNO_3_ solution, and potential scanning was obtained at a scan rate of 50 mV s^−1^ within the applied voltage range of 1.0 to 2.0 V vs. Ag/AgCl for different numbers of electrochemical activation cycles (5, 10, and 20 cycles). The extended graphite with different activation degrees were conducted by using cyclic voltammetry (CV) cycling processes, which can be expected to induce extended defect on the surface of graphite, in this text referred to as extended graphite, providing a new set of growth platforms to form hierarchical flower-like MnO_2_. The obtained extended graphite with three different degree activations (low, medium, and high degree activation) were denoted as gra-L, gra-M, and gra-H, respectively. The obtained extended graphite was then washed with DI water to remove the remaining reagents and collected for subsequent MnO_2_ synthesis. MnO_2_ was deposited on the extended graphite by a spontaneous redox reaction between transition metal ions and carbon materials, which involve simultaneous oxidation of the extended graphite and reduction of KMnO_4_. The increased structure defects of the carbon materials may optimally tune the oxidation potential of carbon materials and thus more easily promote KMnO_4_ reduction to MnO_2_ [17]. The synthesis procedures in detail were as follows: gra-L, gra-M, and gra-H were immersed in 50 mL aqueous solutions of 0.001 M KMnO_4_ with stirring at 80 °C. Subsequently, these obtained products (MnO_2_/extended graphite) were rinsed three times by DI water and dried at 60 °C for 3 h (denoted as MnO_2_/gra-L, MnO_2_/gra-M, and MnO_2_/gra-H, respectively). The resulted products were then collected for the following characterization.

### 2.3. Apparatus

The morphology was characterized by using field emission scanning electron microscopy (FESEM, JSM-7410F, JEOL, Akishima, Japan) and field emission transmission electron microscopy (FETEM, JEM-2100F, JEOL, Akishima, Japan). The chemical structure and composition were determined by X-ray photoelectron spectroscopy (XPS, PHI-5000 Versaprobe, ULVAC-PHI, Tokyo, Japan). Synchrotron X-ray absorption spectroscopy (XAS) at C K-, O K- and Mn L-edges were performed at BL20A of the National Synchrotron Radiation Research Center (NSRRC), Hsinchu, Taiwan. Electrochemical measurements were performed using a three-electrode system comprised of as-prepared sample-graphite paste electrode as current collector (mixing graphite powder and carbon black in 4:1 weight ratio dispersed terpineol) by casting on plastic substrate with an active material mass loading of 0.4 mg cm^−2^, a platinum wire counter electrode; and an Ag/AgCl (3 M KCl) reference electrode by an electrochemical analyzer (Autolab, model PGSTAT30, Eco Chemie, Utrecht, The Netherlands). The electrochemical testing in supercapacitors and electrochemical glucose sensing was performed in the two different aqueous electrolyte solutions as supporting electrolyte (using 1 M Na_2_SO_4_ for supercapacitors and 0.1 M NaOH for electrochemical glucose sensing). In the supercapacitor, the capacitive performance was evaluated thorough cyclic voltammetry (CV) and galvanostatic charge–discharge (GCD) in 1 M Na_2_SO_4_ within the applied voltage range of 0.0 to 1.0 V vs. Ag/AgCl. In electrochemical glucose sensing, the sensing performance of electrochemical sensor was evaluated using CV and amperometry in the 0.1 M NaOH in the absence and presence of glucose. LCMS/MS analysis was conducted using a Q-TOF mass spectrometer (LCMS-9030, Shimadzu Corp., Kyoto, Japan) and a triple quadrupole mass spectrometer (LCMS-8045, Shimadzu Corp., Kyoto, Japan). A Unison UK-Amino column (UKA24U, Imtakt USA, Portland, OR, USA) was utilized for the chromatographic separation.

## 3. Results

**Figure 1:**
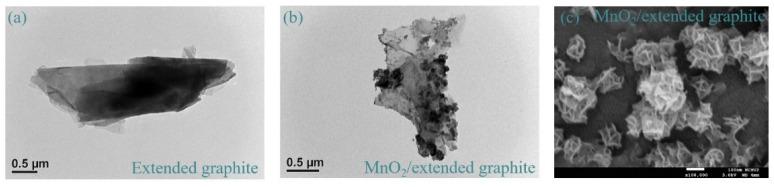
FETEM of (**a**) extended graphite and (**b**) MnO_2_/extended graphite. (**c**) FESEM of MnO_2_/extended graphite.

**Figure 2:**
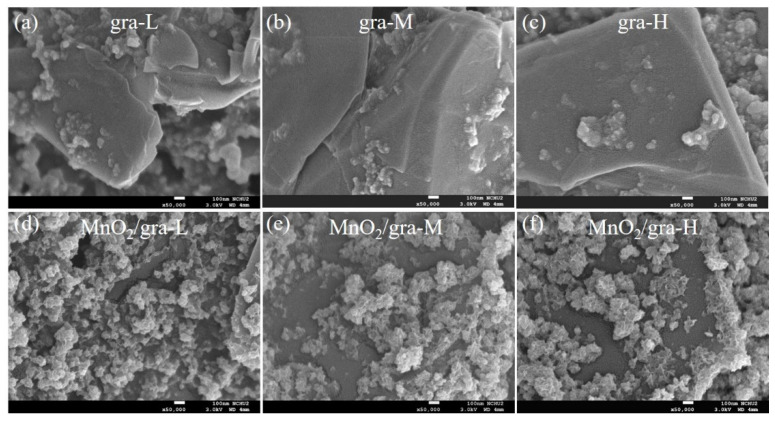
FESEM of (**a**) gra-L, (**b**) gra-M, (**c**) gra-H, (**d**) MnO_2_/gra-L, (**e**) MnO_2_/gra-M, and (**f**) MnO_2_/gra-H.

The morphologies of extended graphite and MnO_2_/extended graphite are characterized by FETEM and FESEM. FETEM images in Figure 1a display the general morphologies and detailed microstructures of extended graphite. It clearly indicates that the surfaces of extended graphite are rough. This result implies that the defect densities in graphite can be fine-tuned through electrochemically activated treatment of graphite in the HNO_3_ solution that effectively expose more edge and basal plane defect sites in the 2D structure graphite layers. This work presents a facile synthesis strategy to synthesize extended defect structures in graphite layers and further introduces functional groups at defect sites along the extended graphite. Extended graphite attached to these defect sites offers an ideal platform for the growth of MnO_2_ in the KMnO_4_ solution by spontaneous redox reaction. Figure 1b,c shows that MnO_2_/extended graphite has a 3D hierarchical flower-like MnO_2_ construction consisting of many assembled ultrathin 2D nanosheets, and it develops an interconnected network within extended graphite, confirming the successful synthesis of the MnO_2_/extended graphite. The detailed structural morphologies of 3D hierarchical flower-like MnO_2_ over the extended graphite with the three different degrees of electrochemically activated treatment (MnO_2_/extended graphite) are represented by EFSEM. Figure 2 displays the EFSEM images of extended graphite with different activation degrees (gra-L, gra-M, and gra-H) and MnO_2_/extended graphite (MnO_2_/gra-L, MnO_2_/gra-M, and MnO_2_/gra-H). It can be clearly seen in Figure 2a–c that the surface morphologies of extended graphite show no obvious differences when the activation degrees are controlled by electrochemically treatment. With extended graphite as an ideal platform for the growth of MnO_2_, the 3D hierarchical flower-like MnO_2_ is grown at the defect structures of extended graphene through in situ nucleation and growth processes (see Figure 2d–f). The extended graphite via electrochemically treatment exposes numerous edge and basal plane defect sites that promote the subsequent nucleation and growth of 3D hierarchical flower-like MnO_2_, especially in view of MnO_2_/gra-H. The FESEM image of MnO_2_/gra-H in Figure 2f shows the conductive gra-H interconnected with the 3D hierarchical flower-like MnO_2_ owing to clear and distinct crumpled layer structure consisting of many assembled ultrathin 2D nanosheets, which is consistent with the TEM images in the Figure 1b. The 3D hierarchical structure of MnO_2_/gra-H constructed by the formation of the interconnected conducting networks between defect sites of extended graphite and ultrathin MnO_2_ nanosheets ensures good electrical contact and also provides a large electrochemically active surface area, which further not only contributes to a continuous electron pathway, but also facilitates ion transport by shortening diffusion pathways. The above results clearly reveal that the 3D hierarchical MnO_2_/extended graphite could serve as functional materials in electrochemical techniques.

**Figure 3:**
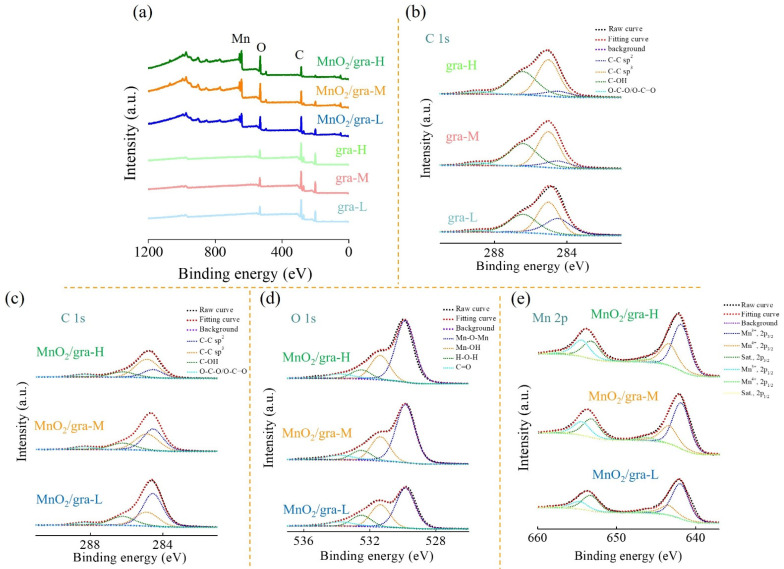
(**a**) Full scan (**b**,**c**) C 1s, (**d**) O 1s, and (**e**) Mn 2p XPS spectra of extended graphite and MnO_2_/extended graphite.

The surface chemical and electronic states of extended graphite and MnO_2_/extended graphite are characterized by XPS and XAS. Figure 3a presents the XPS full survey spectrum of extended graphite and MnO_2_/extended graphite. The results indicate the coexistence of C and O elements in the extended graphite with different activation degrees (gra-L, gra-M, and gra-H). After further growth of 3D hierarchical flower-like MnO_2_ within extended graphite, the full survey spectrum shows signals from C, O, and Mn elements, suggesting the obtained MnO_2_/extended graphite was successfully synthesized. The high-resolution C 1s XPS spectrum of gra-L, gra-M, and gra-H is shown in Figure 3b. The high-resolution C 1s spectrum of gra-L, gra-M, and gra-H (Figure 3b) shows four different peaks located at 284.5, 285.0, 286.4, and 288.9 eV, which correspond to C–C (sp^2^), C–C (sp^3^), C-OH (hydroxyl), and O-C-O/O-C=O (epoxy and carboxyl) functional groups, respectively [18]. The XPS quantitative analysis results are summarized in Table 1, Table 2, Table 3. From Table 1, it is clear that the content of sp^3^ carbon bonds, hydroxyl, epoxy, and carboxyl functional groups within extended graphite are increased due to the destruction of the sp^2^ carbon bonds and the formation of defective sites with increasing activation degree, which proves that extended graphite exists from an electrochemically activated transformation and reflects the relative sp^3^/sp^2^ carbon bonds ratio [19,20]. It is well known that the functional groups are mainly located at the basal and edges planes of graphite, which not only facilitates the exfoliation process of graphite sheets, but also modifies surface hydrophilicity of exfoliate graphite [21,22,23,24]. The extended graphite by electrochemically activated treatment leads to the formation of sp^3^ carbon bonds and creates defect sites, providing numerous nucleation sites that facilitate the uniform nucleation and growth of the selected substances [25,26].

Figure 3c–e show the high-resolution C 1s, O 1s, and Mn 2p XPS spectrum of MnO_2_/extended graphite. The high-resolution O 1s XPS spectrum of the MnO_2_/extended graphite can be deconvoluted into four peaks corresponding to Mn-O-Mn, Mn-O-H, H-O-H, and C=O located at 529.8, 531.4, 532.5, and 533.8 eV. The Mn 2p XPS spectrum of the MnO_2_-coated different carbon materials in Figure 3f consist of two doublet peaks of Mn 2p_3/2_ and 2p_1/2_ located at 642 and 653 eV, with a typical spin energy separation (~11.7 eV) of the Mn 2p doublet peaks. Furthermore, the overlapped Mn 2p_3/2_ (2p_1/2_) spectra can be fitted into three peaks at 641.9 (653.3), 643.4 (654.5), and 645.3 (657.5) eV, which are characteristics of Mn^3+^, Mn^4+^, and shake-up satellite (denoted as Sat.), respectively, further confirming the presence of mixed-valance manganese oxide with oxidation state Mn^3+^ and Mn^4+^ [27,28]. A deeper analysis into the XPS quantitative analysis in the MnO_2_/extended graphite (Table 3) shows that the Mn^3+^/Mn^4+^ in the MnO_2_/extended graphite decreases with MnO_2_ growth on the extended graphite by gradual increase of activation degrees. This suggests that higher content of Mn^4+^ is more pronounced for MnO_2_/gra-H. Second, MnO_2_/extended graphite exhibits an increase in sp^2^ carbon bonds and the decrease in hydroxyl in comparison with that of electrochemically activated extended graphite (see Figure 3c and Table 1 and Table 2), which indicates that the introduction of MnO_2_ within electrochemically activated extended graphite can greatly increase the relative content of sp^2^ carbon bonds, further improving the microcrystalline structure and graphitization degree. It can be expected to display better electrical conductivity and further promote a rapid and efficient charge transfer at the active electrode material/electrolyte interfaces, thus leading to a significantly enhanced electrochemical performance. Third, the decrease in the sp^2^ carbon bonds causes the gradual increase in Mn-O-Mn with the MnO_2_ growth over the three different activation degrees of the extended graphite, especially when MnO_2_ grows on the gra-H due to the more severe activation degree, which exposes abundant defective sites and favors the nucleation and growth of MnO_2_ [29,30,31].

**Figure 4:**
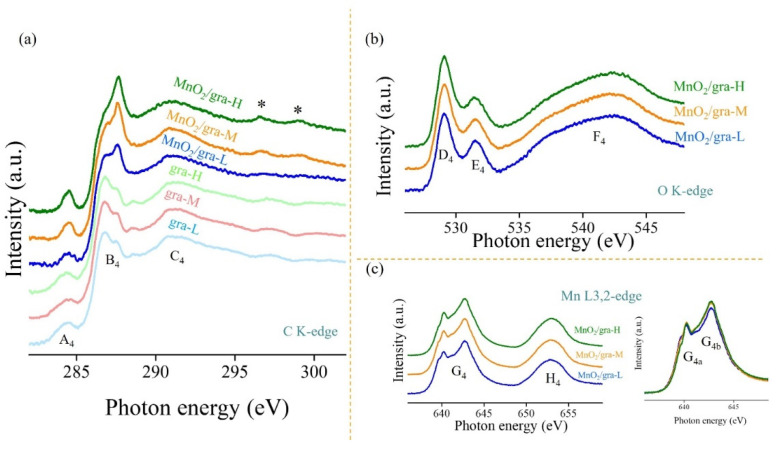
(**a**) C K-edge, (**b**) O K-edge, and (**c**) Mn L3, 2-edge XAS spectra of extended graphite and MnO_2_/extended graphite. Inset in (**c**) is the region of Mn L3-edge XAS spectra. The features (denoted as *) at 298.6 and 301.0 eV are potassium ions and water molecules within the interlayer space of MnO_2_ structure via spontaneous redox reaction in the KMnO_4_ solution.

Figure 4 presents the C K-edge, O K-edge, and Mn L3,2-edge XAS of extended graphite and MnO_2_/extended graphite. XAS is known to be a powerful tool sensitive to understanding the information on local atomic and electronic structure details caused by electronic transition from a core level to various unoccupied states. Figure 4a shows the C K-edge XAS of extended graphite and MnO_2_/extended graphite. Features A_4_, B_4_, and C_4_ are associated with the electronic transition from C 1s to the unoccupied C–C π* (ring) orbitals, the chemically functionalized carbon atoms and/or defects, and unoccupied C–C σ* (ring) orbitals, respectively. With MnO_2_ growth on the extended graphite, the pronounced C−C π* (A_4_) resulting from significant charge-transfer interactions between extended graphite and Mn atoms reveals that MnO_2_/extended graphite has the perfect graphitic crystalline structure of graphite sheets and demonstrates excellent electrical conductivity. Additionally, these charge-transfer interactions between MnO_2_ and electrochemically activated extended graphite induce dramatic changes in Feature B_4_, which pertains to the construction of new Mn−O−C bonds. It is reasonable to believe that the oxygen functional groups on extended graphite can act as anchoring or nucleation sites for the growth of MnO_2_. The results support the fact that the electrochemically activated extended graphite provides an interconnected conducting network, suggesting that these ultrathin MnO_2_ nanosheets are tightly anchored within extended graphite. The features (denoted as *) at 298.6 and 301.0 eV are associated with the K L3- and L2-edge that can be explained by the presence of potassium ions and water molecules generally intercalated within the interlayer space of MnO_2_ structure via spontaneous redox reaction in the KMnO_4_ solution [32], especially in view of MnO_2_/gra-H.

Figure 4b,c show the O K-edge and Mn L3,2-edge XAS of MnO_2_/extended graphite. These O K-edge XAS spectra present similar features (labeled as D_4_, E_4_, and F_4_) from the MnO_2_/extended graphite (MnO_2_/gra-L, MnO_2_/gra-M, and MnO_2_/gra-H). Feature F_4_ can be assigned to electronic transition from O 1s to Mn 4sp transition. Features D_4_ and E_4_ are related to the electronic transition from hybridization of O 2p and metal Mn 3d t_2g_ and 3d e_g_ orbitals. Variation in the three features represents that electrochemically activated extended graphite can possess the Mn-O bonding and the surface configuration, favoring the electronic interactions between MnO_2_ and electrochemically activated extended graphite. The ratio of the intensity of Features D_4_ and E_4_ increases with the MnO_2_ growth within more severe activation degree of extended graphite, which suggests that the surface modification (structural defects and chemical functionalization) of extended graphite affects the nucleation process. It is believed that the surface sufficient functional groups within extended graphite can provide reactive sites for the nucleation and growth of MnO_2_. The Mn L3,2-edge XAS of MnO_2_/extended graphite display doublet features (labeled as G_4_ and H_4_) that arise from the Mn 2p_3/2_ and 2p_1/2_ core level states to the unoccupied Mn 3d states electronic transition, which can be correlated with the valence state of Mn atoms in the MnO_2_ [33]. Notably, Feature G_4_ can be split into two peaks (G_4a_ and G_4b_) (inset in Figure 4c), and the mixed valence states of Mn^3+^ and Mn^4+^ and their ratios within MnO_2_/extended graphite can be estimated from the ratio of G_4a_/G_4b_. A decrease in the G_4a_/G_4b_ ratio with the MnO_2_ growth within more severe activation degrees of extended graphite may give evidence that the MnO_2_/gra-H contains Mn in a higher oxidation state [34,35]. These XAS results are consistent with the XPS results in Figure 3.

Hence, the overall analysis of the XPS and XAS results demonstrate that extended graphite by electrochemically activated treatment can create defect sites and provide abundant nucleation sites for the growth of MnO_2_; in the meantime, MnO_2_/extended graphite favorably attain the sp^2^ carbon bonds character in graphite sheet, resulting in better electrical conductivity. It is concluded that MnO_2_/extended graphite could be a good candidate for the future design of synergistic multifunctional materials in electrochemical techniques. In this study, MnO_2_/extended graphite are synthesized by spontaneous redox reaction and used as bifunctional electrode materials for supercapacitors and glucose sensing.

**Figure 5:**
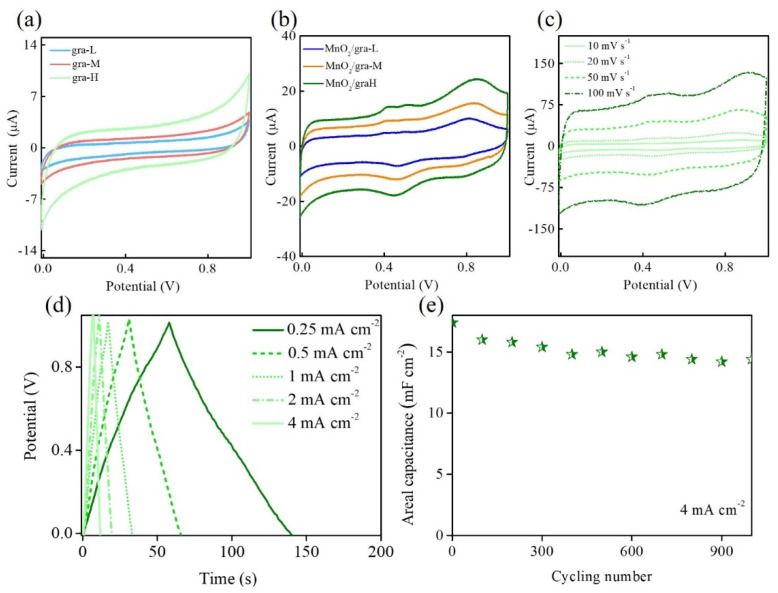
CV curves of (**a**) gra-L, gra-M, and gra-H, and (**b**) MnO_2_/gra-L, MnO_2_/gra-M, and MnO_2_/gra-H in 1 M Na_2_SO_4_ at a scan rate of 20 mV s^−1^. (**c**) CV curves of MnO_2_/gra-H in 1 M Na_2_SO_4_ at different scan rates. (**d**) Galvanostatic charge/discharge curves of MnO_2_/gra-H in 1 M Na_2_SO_4_ at different current densities. (**e**) Cyclic stability of MnO_2_/gra-H in 1 M Na_2_SO_4_ is recorded every 100 cycles during cycling (denoted by green star).

For the potential use of MnO_2_/extended graphite in supercapacitors, Figure 5 shows the electrochemical capacitive performance of extended graphite and MnO_2_/extended graphite thorough cyclic voltammetry (CV) and galvanostatic charge–discharge (GCD) in 1 M Na_2_SO_4_. In comparison to the CV results of extended graphite and MnO_2_/extended graphite in Figure 5a,b, these results clearly reveal that extended graphite (gra-L, gra-M, and gra-H) have greater electrical double-layer capacitive behavior with more severe activation degree, which might be attributed to the functionalized and defective extended graphite owing to these strong interfacial interactions. Thus, it could be expected that increasing the interfacial area and interfacial interactions within extended graphite helps build an intimate interface to facilitate interactions between extended graphite and KMnO_4_ interfaces for MnO_2_ growth by spontaneous redox reaction. Figure 5b shows that the CV curve of MnO_2_/extended graphite gives a quasi-rectangular shape with two pairs of well-defined redox peaks corresponding to both the functionalized extended graphite and the Mn^3+^/Mn^4+^ redox reaction. Figure 4a reveals that controlled growth of 3D hierarchical flower-like MnO_2_ within extended graphite results in more perfect crystalline structure of the graphitic carbon. MnO_2_/extended graphite, because of its perfect graphitic crystalline structure, should possess good conductivity to effectively ensure electron transport, and hence the redox reaction can proceed more effectively and reversibly; there most likely exists two distinct pseudocapacitance charge-storage mechanisms. It demonstrates that the synergistic effect of extended graphite and MnO_2_ results in the coexistence of both the electrical double-layer capacitance of extended graphite and pseudocapacitance of extended graphite and MnO_2_. Hence, the CV result in Figure 5b indicates that MnO_2_/gra-H displays better capacitive performance compared to MnO_2_/gra-M and MnO_2_/gra-L. Thanks to the excellent surface modification of gra-H, there is a perfect graphitic crystalline structure present in MnO_2_/gra-H, which provides a continuous conductive network to optimize electron/ion transport kinetics for boosting the electrochemical performance. Figure 5c displays the CV curves of MnO_2_/gra-H measured at different scan rates of 10, 20, 50, and 100 mV s^−1^. The CV curves maintain their shape and reversibility even at relatively high scan rates, suggesting excellent capacitive performance of MnO_2_/gra-H. The GCD measurements are used for further discussing the enhanced capacitive performance of MnO_2_/gra-H (Figure 5d). MnO_2_/gra-H displays quasi-triangular symmetrical shape by GCD measurements at different current densities of 0.25, 0.5, 1, 2, and 4 mA cm^−2^, and indicates the coexistence of both electrical double-layer capacitance and pseudocapacitance, which are consistent with the CV curves. The areal capacitance values of MnO_2_/gra-H can be calculated according to the following equation: C = (I Δt)/(ΔV A), where C is areal capacitance (mF cm^−2^), I is the charge/discharge current (mA), Δt is the discharge time (s), ΔV is the voltage change during discharge (V), and A is the area of the electrode (cm^2^). The areal capacitance (gravimetric capacitance) values at 0.25, 0.5, 1, 2, and 4 mA cm^−2^ current density are calculated to be 20.4, 16.8, 16.0, 15.8, and 15,7 mF cm^−2^ (51, 42.0, 40.0, 39.5, and 39.3 F g^−1^), respectively. The capacitive performance of MnO_2_/gra-H is comparable to other previously reported results in transition metal-based electrode materials (Table 4) [36,37,38,39,40]. In order to further research the long-term cycling stability of the MnO_2_/gra-H, MnO_2_/gra-H is performed over 1000 consecutive galvanostatic charge–discharge cycles at a constant current density of 4 mA cm^−2^ (Figure 5e). After 1000 cycles, the capacitance is equivalent to 83% of initial value, demonstrating a good cycling performance. These results once again demonstrated that the MnO_2_/extended graphite consisting of the interconnected conducting networks between 3D hierarchical flower-like MnO_2_ and extended graphite provide tight contact and stable interface, which facilitates an enhanced capacitive performance for supercapacitors.

**Figure 6:**
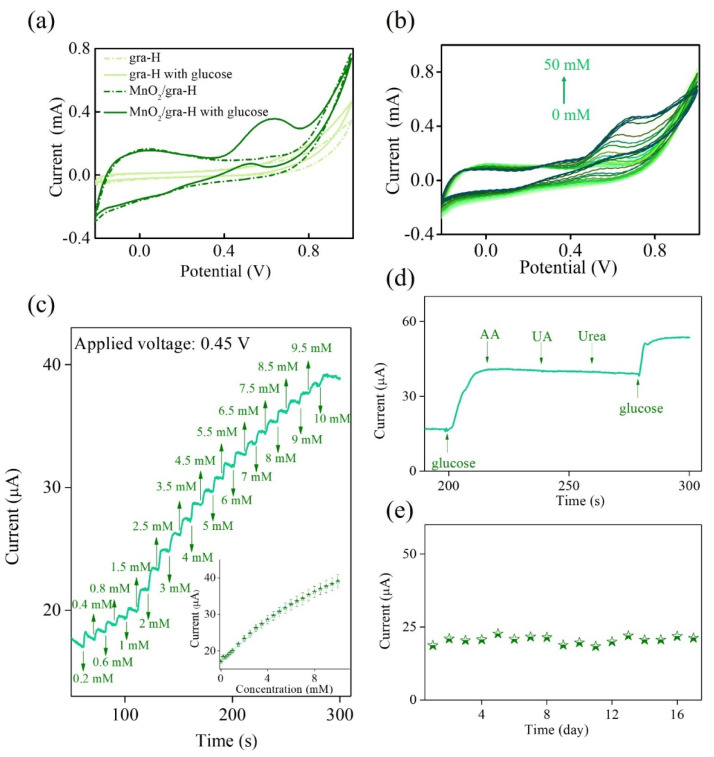
(**a**) CV curves of gra-H and MnO_2_/gra-H in 0.1 M NaOH in the absence (dashed lines) and presence (solid lines) of 10 mM glucose at a scan rate of 50 mV s^−1^. (**b**) CV curves of MnO_2_/gra-H in 0.1 M NaOH with successive addition of various glucose concentrations. (**c**) Amperometry of MnO_2_/gra-H in 0.1 M NaOH with successive addition of various glucose concentrations at applying voltage +0.45 V. In inset of (**c**) is the corresponding calibration plot of the response current versus glucose concentration. (**d**) Interference tests and (**e**) long-term stability of MnO_2_/gra-H in 0.1 M NaOH at applying voltage +0.45 V (denoted by green star).

It is anticipated that the findings of this work could open another opportunity to explore its possible applications in non-enzymatic glucose sensing. Figure 6 displays the electrochemical sensing performance of gra-H and MnO_2_/gra-H using cyclic voltammetry (CV) and amperometry towards glucose electrooxidation in alkaline solution. In Figure 6a, gra-H and MnO_2_/gra-H are examined using CV under the scan rate of 50 mV s^−1^ in 0.1 M NaOH in the absence and presence of 10 mM glucose. The successive addition of glucose results in an increase in the anodic (oxidation) current at 0.4~0.8 V. Specifically, the anodic (oxidation) peak current of glucose remarkably increases at MnO_2_/gra-H. This result reveals that the MnO_2_/gra-H has great potential to electrocatalyze glucose oxidation reactions effectively involving the redox-active Mn mediated electrocatalytic reaction mechanisms [41]. The mechanisms can be illustrated as follows.

Mn^3+^ + OH^−^ ↔ Mn^4+^ + H_2_O + e^−^ (1)

Mn^4+^ + glucose + e^−^ → Mn^3+^ + gluconolactone (2)

When increasing the glucose concentration in the range from 0 to 50 mM, MnO_2_/gra-H exhibits a gradual increase anodic (oxidation) peak current, demonstrating that MnO_2_/gra-H provides cooperative effects in promoting the electrocatalytic activity toward glucose electrooxidation. Furthermore, there is continuously a progressive positive shift in anodic (oxidation) peak potential at a voltage region of 0.4∼0.8 V, which is caused by a kinetic limitation in the glucose electrooxidation between redox sites within MnO_2_/gra-H (Figure 6b) [42]. Figure 6c shows the amperometry of MnO_2_/gra-H towards glucose electrooxidation in 0.1 M NaOH at 0.45 V, with the gradual additions of glucose concentrations up to 10 mM. The inset of Figure 6c shows the corresponding calibration plot of the response current (I) versus glucose concentration (Conc._glu_) (error bars represent the standard deviations for the 3-times repeated measurements). Response current increases linearly with increasing glucose concentration up to about 5 mM. The corresponding calibration equation is I (mA) = 0.003 Conc._glu_ (mM) + 0.017, having the resulting correlation coefficient value (R^2^) 0.994, sensitivity 43 μA mM^−1^cm^−2^, and the limit of detection 0.081 mM based on a signal-to-noise ratio of 3 (S/N = 3), indicating the significantly improved electrocatalytic activity of MnO_2_/gra-H for glucose electrooxidation. The present MnO_2_/gra-H exhibits better or comparable performance with some previously reported results with similar electrode materials [43,44,45,46]. The comparison results are summarized in Table 5. The interference test is conducted by amperometry in 0.1 M NaOH at 0.45 V with the gradual additions of 4.83 mM glucose, 0.05 mM ascorbic acid (AA), 0.5 mM uric acid (UA), 5 mM urea, and 4.83 mM glucose, which does not cause any observable interference signal to glucose detection (Figure 6d). This means that the proposed MnO_2_/gra-H possesses excellent selectivity. It is well known that the long-term stability is also an important index to evaluate the sensing performance. Hence, the long-term stability of the proposed MnO_2_/gra-H is evaluated by measuring its amperometric current response to 1 mM glucose every day for 2 weeks. Figure 6e reveals that the amperometric current response stabilizes to approximately 80~90% of its initial value, implying an excellent stability during long-term storage.

To further demonstrate the potential of the proposed MnO_2_/gra-H in practical applications, the recovery tests of glucose are carried out by adding a known concentration (1 mM) to samples, which is then evaluated by analyzing the amperometric response to glucose by 3-time repeated measurements. The results of the recovery tests obtained by the proposed amperometric method are in good agreement with the results obtained by the reference method (mass spectrometry), and the comparison results are given in Table 6. The recovery test demonstrates that the proposed MnO_2_/gra-H makes satisfactory recovery of the added glucose concentrations.

## 4. Conclusions

In this study, MnO_2_/extended graphite displayed high bifunctional electro-activity performances in electrochemical multifunctional applications (including supercapacitors and glucose sensing). Thanks to the excellent surface modification of gra-H through electrochemically activated treatment of graphite inducing extended defects on the surface of graphite, providing a new set of growth platforms to form hierarchical flower-like MnO_2_, and thus a perfect graphitic crystalline structure present in MnO_2_/gra-H, which provide a continuous conductive network to optimize electron/ion transport kinetics for boosting the electrochemical performances of supercapacitors (areal capacitance value of 20.4 mF cm^−2^ at 0.25 mA cm^−2^ and capacitance retention of 83% after 1000 cycles) and glucose sensing (linear range up to about 5 mM, sensitivity of 43 μA mM^−1^cm^−2^, and the limit of detection of 0.081 mM). Because of its electrochemical synergistic effects, MnO_2_/extended graphite is a promising candidate for the development of electrochemical multifunctional applications.

## Figures and Tables

**Table 1:** Fitted results of C 1s XPS spectra.

Samples	Fitted Results of C 1s XPS Spectra			
	C-C sp^2^ (%)	C-C sp^3^ (%)	C-OH (%)	O-C-O/O-C=O (%)
gra-L	28.3	37.0	32.5	2.2
gra-M	12.6	42.4	41.4	3.6
gra-H	10.1	43.9	42.3	3.7
MnO_2_/gra-L	53.5	23.5	19.3	3.7
MnO_2_/gra-M	42.9	35.3	17.0	4.8
MnO_2_/gra-H	22.5	52.3	18.8	6.4

**Table 2:** Fitted results of O 1s XPS spectra.

Samples	Fitted Results of O 1s XPS Spectra			
	Mn-O-Mn (%)	Mn-OH (%)	H-O-H (%)	C=O (%)
MnO_2_/gra-L	51.1	26.0	13.5	9.4
MnO_2_/gra-M	59.5	22.9	9.5	8.1
MnO_2_/gra-H	61.5	22.2	8.5	7.8

**Table 3:** Fitted results of Mn 2p XPS spectra.

Samples	Fitted Results of Mn 2p XPS Spectra						
	Mn^3+^ 2p_3/2_ (%)	Mn^3+^ 2p_1/2_ (%)	Mn^4+^ 2p_3/2_ (%)	Mn^4+^ 2p_1/2_ (%)	Sat. 2p_3/2_ (%)	Sat. 2p_1/2_ (%)	Mn^3+^/Mn^4+^ Ratio
MnO_2_/gra-L	47.1	20.2	17.2	10.3	3.1	2.1	2.4
MnO_2_/gra-M	41.9	18.2	20.3	14.2	3.2	2.0	1.7
MnO_2_/gra-H	39.4	16.2	22.7	16.4	3.1	2.2	1.4

**Table 4:** Comparison of the capacitive performance with transition metal-based electrode materials.

Electrode Materials	Electrolyte	Current Density	Areal Capacitance (mF cm^−2^)	Reference
Mn_3_N_2_	Na_2_SO_4_	1.00 mA cm^−2^	74.0	[36]
MnO_x_	Na_2_SO_4_	0.25 mA cm^−2^	19.3	[37]
TiO_2_	Na_2_SO_4_	2.00 mV s^−1^	23.2	[38]
NiCo_2_O_4_	KOH	0.25 mA cm^−2^	28.0	[39]
ZnCo_2_O_4_	KOH	0.01 mA cm^−2^	16.1	[40]
MnO_2_/gra-H	Na_2_SO_4_	0.25 mA cm^−2^	20.4	This work

**Table 5:** Comparison of the non-enzymatic glucose sensing performance.

Electrode Materials	Applied Potential (V vs. Ag/AgCl)	Linear Range (mM)	Sensitivity (μA mM^−1^cm^−2^)	Detection Limit (mM)	Reference
MnCo–carbon nanofibers/Nafion	0.60	0.5–7.0	36	0.050	[43]
PtAu–MnO_2_	0.00	0.1–30.0	59	0.020	[44]
MnCu/MWCNT/GO	−0.05	1.0–32.0	59	0.001	[45]
PDDA-RGO/MnO_2_/AuNPs	0.60	0.02–0.85	84	0.002	[46]
MnO_2_/gra-H	0.45	Up to 5.0	43	0.080	This work

**Table 6:** The results of the recovery tests obtained by the electrochemical sensing and the mass spectrometric methods for glucose.

Added (mM)	Found by Electrochemical Sensing (mM)	Recovery (%)	RSD (%)	Found by Mass Spectrometry (mM)	Recovery (%)	RSD (%)
1	1.02	102	5.2	0.94	94	0.9
